# Exercise therapy for bone and muscle health: an overview of systematic reviews

**DOI:** 10.1186/1741-7015-10-167

**Published:** 2012-12-19

**Authors:** Kåre Birger Hagen, Hanne Dagfinrud, Rikke Helene Moe, Nina Østerås, Ingvild Kjeken, Margreth Grotle, Geir Smedslund

**Affiliations:** 1 National Resource Centre for Rehabilitation in Rheumatology, Department of Rheumatology, Diakonhjemmet Hospital, P.O. Box 23 Vinderen, 0319 Oslo, Norway

**Keywords:** fibromyalgia, low back pain, neck pain, shoulder pain, osteoarthritis, rheumatoid arthritis, ankylosing spondylitis, osteoporosis, pain, physical function

## Abstract

**Background:**

Musculoskeletal conditions (MSCs) are widely prevalent in present-day society, with resultant high healthcare costs and substantial negative effects on patient health and quality of life. The main aim of this overview was to synthesize evidence from systematic reviews on the effects of exercise therapy (ET) on pain and physical function for patients with MSCs. In addition, the evidence for the effect of ET on disease pathogenesis, and whether particular components of exercise programs are associated with the size of the treatment effects, was also explored.

**Methods:**

We included four common conditions: fibromyalgia (FM), low back pain (LBP), neck pain (NP), and shoulder pain (SP), and four specific musculoskeletal diseases: osteoarthritis (OA), rheumatoid arthritis (RA), ankylosing spondylitis (AS), and osteoporosis (OP). We first included Cochrane reviews with the most recent update being January 2007 or later, and then searched for non-Cochrane reviews published after this date. Pain and physical functioning were selected as primary outcomes.

**Results:**

We identified 9 reviews, comprising a total of 224 trials and 24,059 patients. In addition, one review addressing the effect of exercise on pathogenesis was included. Overall, we found solid evidence supporting ET in the management of MSCs, but there were substantial differences in the level of research evidence between the included diagnostic groups. The standardized mean differences for knee OA, LBP, FM, and SP varied between 0.30 and 0.65 and were significantly in favor of exercise for both pain and function. For NP, hip OA, RA, and AS, the effect estimates were generally smaller and not always significant. There was little or no evidence that ET can influence disease pathogenesis. The only exception was for osteoporosis, where there was evidence that ET increases bone mineral density in postmenopausal women, but no significant effects were found for clinically relevant outcomes (fractures). For LBP and knee OA, there was evidence suggesting that the treatment effect increases with the number of exercise sessions.

**Conclusions:**

There is empirical evidence that ET has beneficial clinical effects for most MSCs. Except for osteoporosis, there seems to be a gap in the understanding of the ways in which ET influences disease mechanisms.

## Background

Musculoskeletal conditions (MSCs) are common, and have important consequences for the individual and for society. MSCs are the most common cause of severe long-term pain and physical disability, and in Europe, 20 to 30*% *of adults are affected at any one time [1-3]. It is estimated that MSCs represent nearly 25*% *of the total cost of illness in Sweden [4], and they are one of the most common causes of health problems limiting work ability [5]. About one in five consultations in primary care are for MSCs, and some of these patients are referred to other health professionals such as physiotherapists, occupational therapists, or chiropractors, to medical specialists such as rheumatologists, or to orthopedic surgeons. The burden that MSCs create has been recognized by the United Nations and WHO, with their endorsement of the Bone and Joint Decade 2000 to 2010 [6]. The prevalence of many of these conditions increases markedly with age, and many patients also have some common lifestyle factors (obesity, smoking, and physical inactivity). With the increasing number of older people and the ongoing changes in lifestyle, the burden of MSCs and other non-communicable diseases is predicted to increase [7].

MSCs form a heterogeneous group of over 200 different health problems that are linked anatomically and also linked by their association with pain and impaired physical function [8]. They range from conditions of acute onset and short duration to lifelong disorders. For many common MSCs such as regional pain syndromes, the underlying pathogenesis is poorly understood, and it is often not possible to produce a clear-cut diagnosis. Some, such as osteoarthritis (OA), are biologically well defined but clinically less well understood, whereas others, such as rheumatoid arthritis (RA) are both biologically and clinically well defined [9].

For decades, inactivity and bed rest were the mainstays of the management of many diseases, but there is now an increasing amount of evidence supporting the opposite view; that is, that physical activity and exercise are beneficial to health promotion and treatment. Although exercise and physical activity are closely related constructs, they have distinct meanings. The term 'physical activity' includes everyday activities that can contribute to wellbeing, whereas 'exercise' is physical activity that is planned, structured and repetitive [10]. The focus of this overview is exercise therapy (ET), which involves the prescription of a physical-activity program that involves the client undertaking voluntary muscle contraction and/or body movement with the aim of relieving symptoms or improving function, or of improving, retaining, or slowing deterioration of health [11].

Whereas systematic reviews of randomized trials usually summarize the evidence of one kind of intervention for a single condition, overviews of systematic reviews can summarize evidence from more than one systematic review of the same intervention for different conditions. There is, to our knowledge, only one published overview of systematic reviews that has focused on the effects of exercises for MSCs [12]. This overview was based on reviews published up to July 2007, and did not include specific diseases such as osteoporosis and inflammatory joint diseases. Thus, the most updated evidence may not be included in this overview. The main aim of the present overview was to synthesize evidence from systematic reviews on the effects of ET on pain and physical function for eight selected MSCs. In addition, we explored the evidence for the effect of ET on disease pathogenesis, and whether particular components of exercise programs are associated with the size of the treatment effects.

## Methods

### Criteria for considering reviews for inclusion

For this overview, we included systematic reviews on the effects of ET for four common conditions (FM, low back pain (LBP), neck pain (NP), and shoulder pain (SP)) and four specific musculoskeletal diseases (OA, RA, ankylosing spondylitis (AS), and osteoporosis). Other related conditions and musculoskeletal malformations and traumas were not included in this overview. All types of land-based ET interventions were considered.

### Search methods for the identification of reviews

The first search, of the Cochrane database of systematic reviews, was performed in March 2012. All Cochrane reviews on exercises for the aforementioned MSCs were considered for inclusion. Two authors (GS and KBH) assessed the eligibility of reviews based on the inclusion criteria presented above. Cochrane reviews with the most recent update of January 2007 or before were not included, and we replaced these reviews by searching for non-Cochrane systematic reviews in MEDLINE, EMBASE, CINAHL, AMED, and PEDro, published after this date (for the electronic search strategy for MEDLINE, see Additional file 1). Two authors (GS and KBH) then screened these records for new reviews. If several reviews fulfilled the inclusion criteria, we included only one review per combination of disease, intervention, and outcome by choosing the newest review of high quality. If two or more reviews were equal for these criteria, we chose the review with the highest number of primary studies. We also searched in the same databases for reviews with the explicit aim of investigating the effect of exercise on the pathogenesis of MSCs, with no restrictions on publication date.

### Outcome measures

ET. can have clinical effects by improving dominant symptoms, and we therefore chose to primarily focus on the core symptoms of MSCs: 1) any measure of pain, and 2) physical disability or physical function.

In addition, we collected data on the effect of ET on disease pathogenesis, and whether particular components of exercise programs are associated with the magnitude of treatment effects.

We did not focus on the general health benefits or complications of exercise because small to medium randomized controlled trials (RCTs) with short-term to intermediate follow-up may be inadequate to assess other health benefits such as the cardiovascular risk profile and the incidence of complications. Other potentially relevant outcomes, such as work participation and health-related quality of life, compliance, or costs, were not evaluated.

### Data collection and analysis

One reviewer (GS) extracted data on population, intervention, comparison, and outcomes (inclusion criteria) and the methodological quality of included trials. The methodological quality was assessed using the AMSTAR (A Measurement Tool to Assess Systematic Reviews) checklist [13]. The 11 criteria shown in Table 1 were rated as 'met,' 'unclear/partly met,' or 'not met'. A second reviewer (KBH) independently verified the accuracy of the numeric results. Most reviews included several comparisons, and we included analyses that compared any exercise intervention with no or minimal exercise intervention. Because pain and function most often are continuous outcomes, data were summarized using the standardized mean difference (SMD) or weighted mean difference (WMD) with 95*% *confidence interval (95*% *CI) as reported in the included reviews. For dichotomous outcomes, odds ratio and 95*% *CI were presented. Pooled effect estimates were presented according to the model used in the reviews. For reviews that explored whether particular components of exercise programs were associated with the magnitude of treatment effects, we extracted the coefficients from meta-regression or sub-group analyses. Results from the review investigating the evidence for the effect of ET on disease pathogenesis were reported as in the original review.

**Table 1:** The AMSTAR check list for methodological assessment.

No	Item
1	Was an 'a priori' design provided?
2	Was there duplicate study selection and data extraction?
3	Was a comprehensive literature search performed?
4	Was the status of publication (that is, 'gray' literature) used as an inclusion criterion?
5	Was a list of studies (included and excluded) provided?
6	Were the characteristics of the included studies provided?
7	Was the scientific quality of the included studies assessed and documented?
8	Was the scientific quality of the included studies used appropriately in formulating conclusions?
9	Were the methods used to combine the findings of studies appropriate?
10	Was the likelihood of publication bias assessed?
11	Were potential conflicts of interest included?

## Results

We identified 18 potentially relevant Cochrane systematic reviews. After reading the full text reviews, two were excluded because they did not focus on land-based ET, two because of irrelevant outcomes, four because of other diagnoses, and finally four because they had not been updated after January 2007 (for the excluded Cochrane reviews and reason for exclusion, see Additional file 2). The search for non-Cochrane reviews after 2007 resulted in 963 references (474 for LBP, 186 for SP, 303 for NP). Of these 963 references, 951 were excluded after screening of the title and abstract and, 12 were retrieved as full text; based on methodological quality and publication date 3 of the latter were included.

### Description of included reviews

We included six Cochrane systematic reviews on patients with FM [14], OP [15], knee OA [16], hip OA [17], RA [18] and AS [19]. In addition, three non-Cochrane reviews comprising patients with SP [20], NP [21] and LBP [22] were included. The nine included reviews comprised at total of 224 trials with 24,059 patients. Characteristics of the included reviews are shown in Table 2.

**Table 2:** Characteristics of included reviews.

Diagnosis [Reference]	Date assessed as up-to-date review	Patient inclusion criteria	Included trials/patients	Methodological quality of included trials	**Methodological quality of reviews **[13]
Fibromyalgia [14]	August 2007	A confirmed diagnosis of fibromyalgia	34/2276	Mean van Tulder scores^a^: 7.33 for aerobic training; 4 for strength training)	Met: all

Low back pain [22]	August 2008	Non-specific low back pain (with or without leg pain) 12 weeks or longer	41/4485	Mean PEDro scores^b^: 6.33 for exercise versus no treatment), and 5.82 for exercise versus minimal intervention	Met: 2, 3, 4, 5, 6, 7, 8 and 9; not met: 10, 11; unclear: 1

Neck pain [21]	February 2008	Non-specific neck pain in adults	33/6218	Mean PEDro scores: 5.67	Met: 2, 6, 7, 11; not met: 3, 4, 5, 8, 9, 10; unclear: 1

Shoulder pain [20]	March 2011	Primary symptom of shoulder pain in adults	17/1436	Mean PEDro scores: 5.87	Met: 5, 7, 11; not met: 3, 4, 8, 10; unclear: 1, 2, 6, 9

Osteoporosis [15]	December 2010	Healthy postmenopausal aged women between 45 and 70 years	43/4320	Most trials were scored as 'unclear' for randomization, allocation concealment, attrition, selective reporting, and assessor blinding	Met: all

Osteoarthritis (knee) [16]	December 2007	Knee OA based on accepted criteria or self-reported knee OA on the basis of chronic joint pain	32/3616	Of 43 trials, 9 (28 *% *) studies had low risk of bias	Met: 1, 2, 4, 5, 6, 7, 8, 9, 11; not met: 3, 10

Osteoarthritis (hip) [17]	August 2008	Hip OA based on accepted criteria or self-reportedhip OA	5/267	Of 5 trials, 3 had low risk of bias and 2 trials had moderate risk of bias	Met: 1, 2, 4, 5, 6, 7, 8, 9, 11; not met: 3, 10

Rheumatoid Arthritis [18]	December 2008	Adults with classic or definite RA based on established criteria	8/575	4 of 8 trials fulfilled at least 8 of 10 methodological criteria	Met: all

Ankylosing spondylitis [19]	January 2007	AS based on the modified New York criteria	11/763	Of 11 trials, 3 had low risk of bias; 3 had moderate risk of bias, and 5 had high risk of bias	Met: all

^a^van Tulder quality scores for internal validity (0 to 11 scale).

^b^PEDro scale scores methodological quality with 11 items.

Four reviews [14,15,18,19] were assessed to be of high methodological quality (all 11 criteria met), whereas in three reviews [16,17,22] eight to nine criteria were met. Finally, in two reviews [20,21] only three to four criteria were met. In addition, we included one review which investigated the evidence for the effect of ET on disease pathogenesis [23].

### Effects of exercises

The effects on clinical outcomes are summarized in Table 3 (pain) and Table 4 (physical functioning).

**Table 3:** Effect of exercise therapy on pain.

Diagnosis [Reference]	Intervention	Control	Included trials/patients	Treatment effect
Fibromyalgia [14]	Aerobic training	Various (wait list, control, no exercise, treatment as usual)	3/183	SMD^a ^0.65 (95*% *CI -0.09 to 1.39)
	
	Strength training	Control (not specified)	1/21	SMD 3.00 (95*% *CI 1.68 to 4.32)

Low back pain [22]	Exercise (many different types)	No treatment	5/NR^b^	WMD^c ^-9.27 on a 100-point scale (95*% *CI-17.00 to -1.55)
		
		Minimal care	11/NR	WMD -4.83 on a 100-point scale (95*% *CI -9.36 to -0.30)

Neck pain [21]	General strength and conditioning exercises	Minimal or no intervention	3/76	WMD -12.00 on a 100-point scale (95*% *CI -22.00 to -2.00)

Shoulder pain [20]	Graded exercise therapy, progressive resistance, active range of motion	Active or no intervention	3/273	SMD -0.30 (95*% *CI -0.48 to -0.12)

Osteoporosis [15]	Static, dynamic, or non-weight-bearing exercise	Various	NR	NR

Osteoarthritis knee [16]	Any land-based therapeutic exercise regimens	Various (not specified)	32/3616	SMD -0.40 (95*% *CI -0.50 to -0.30)

Osteoarthritis hip [17]	Any land-based therapeutic exercise regimens	Various (not specified)	5/204	SMD -0.33 (95*% *CI -0.84 to 0.17)

Rheumatoid arthritis [18]	Short-term aerobic capacity and muscle-strength training	Various (not specified)	1/50	SMD -0.53 (95*% *CI -1.09 to 0.04)
	
	Short-term aerobic capacity training only	Various (not specified)	1/56	SMD -0.27 (95*% *CI -0.79 to 0.26)

Ankylosing spondylitis [19]	Supervised home exercise program	No intervention	1/155	SMD 0.49 (95*% *CI 0.17 to 0.81)

^a ^Standardized mean difference.

^b^Not reported.

^c^Weighted mean difference.

**Table 4:** Effect of exercise therapy on physical functioning.

Diagnosis [Reference]	Intervention	Control	No trials/patients in analyses	Treatment effect
Fibromyalgia [14]	Aerobic training	Various (wait list, control, no exercise, treatment as usual)	4/253	SMD^a ^0.66 (95*% *CI 0.41 to 0.92)
	
	Strength training	Control (not specified)	2/47	SMD 0.52 (95*% *CI -0.07 to 1.10)

Low back pain [22]	Exercise (many different types)	No treatment	9/NR^b^	WMD^c ^-3.31 on a 100-point scale (95*% *CI -4.83 to -1.79)
		
		Minimal care	14/NR	WMD -6.41 on a 100-point scale (95*% *CI -9.76 to -3.05)

Neck pain [21]	General strength and conditioning exercises	Minimal or no intervention	2/141	WMD 1.00 on a 100-point scale (95*% *CI -3.00 to 5.00)

Shoulder pain [20]	Graded exercise therapy, progressive resistance, active range of motion	Active or no intervention	4/358	SMD 0.15 (95*% *CI 0.01 to 0.29)

Osteoporosis [15]	Static, dynamic, or non-weight-bearing exercise	Various	NR	NR

Osteoarthritis knee [16]	Any land-based therapeutic exercise regimen	Various (not specified)	32/3719	SMD -0.37 (95*% *CI -0.49 to -0.25)

Osteoarthritis hip [17]	Any land-based therapeutic exercise regimen	Various (not specified)	5/187	SMD -0.10 (95*% *CI -0.51 to 0.32)

Rheumatoid arthritis [18]	Short-term aerobic capacity and muscle-strength training	Various (not specified)	2/74	SMD -0.40 (95*% *CI -0.86 to 0.06)
	
	Short-term aerobic capacity training only	Various (not specified)	2/66	SMD -0.03 (95*% *CI -0.46 to 0.51)

Ankylosing spondylitis [19]	Supervised home exercise program	No intervention	1/155	SMD 0.12 (95*% *CI -0.20 to 0.43)

^a ^Standardized mean difference.

^b ^Not reported.

^c ^Weighted mean difference.

### Fibromyalgia

FM is characterized by persistent widespread musculoskeletal pain and tenderness. In clinical practice, chronic generalized musculoskeletal pain in all four quadrants of the body that cannot be traced to a specific structural or inflammatory cause is often diagnosed as FM. FM is also associated with other symptoms such as fatigue, stiffness, mood disturbance, abdominal pain, and headache. The 1990 American College of Rheumatology (ACR) diagnosis of FM was based on a history of widespread pain for a duration of longer than 3 months, and the presence of excessive tenderness on applying pressure to 11 of 18 specific muscle-tendon sites, whereas the preliminary 2010 ACR criteria are based on a combination of widespread pain and multi-symptom severity, and do not require a physical or tender-point examination [24].

The Cochrane review of Busch *et al. *[14] compared first aerobic-only exercise interventions (only protocols of intensity recommended by the American College of Sports Medicine) with untreated control groups. Statistical pooling based on three trials with 183 patients indicated a positive non-significant effect on pain in favor of exercise (SMD = 0.65; 95*% *CI -0.09 to 1.39) and a significant effect in physical function (3 trials with 253 patients) (SMD 0.66; 95*% *CI 0.41 to 0.92). Later, meta-analyses from two low-quality trials compared strength-only exercise interventions with untreated control groups, and found a large and significant effect on pain (based on one trial with twenty-one patients) (SMD 3.00; 95*% *CI 1.68 to 4.32), and a non-significant effect in physical function: (SMD 0.52; 95*% *CI -0.07 to 1.10). Based on the number and quality of included trials, Bush *et al. *concluded that there was evidence of a moderate quality showing that short-term aerobic training at the intensity recommended for increases in cardiorespiratory fitness have beneficial effects on physical function and possibly pain. We were not able to identify any analyses exploring the effects of particular components of exercise. Pedersen and Saltin [23] found limited evidence for the effect of exercise on pathogenesis, that is, at least one relevant study of moderate quality is available.

### Low back pain

LBP is generally defined as pain located between the lowest ribs and the inferior gluteal folds, with or without pain radiation to the legs [25]. Clinical guidelines classify LBP into 1) 'non-specific' LBP, 2) LBP affecting the nerve root, and 3) possible serious spinal pathology ('red flags') [26]. After 1 month with LBP symptoms, a specific cause can be identified for approximately 15% of patients [25]. Clinically, patients with LBP form a heterogeneous group presenting with pain of varying duration, and disability in terms of impairment of body function and structures, limitations on activity, and restrictions on participation in work and leisure.

The review by Ferreira *et al. *[22] sought to establish the effect of exercise on pain and disability in patients with chronic non-specific LBP, with the main aim of explaining heterogeneity between trials. For exercise versus minimal care (11 trials), they found a significant weighted mean difference (WMD) of -4.83 (on a 0 to 100 scale) favoring exercise for pain, (95*% *CI -9.36 to -0.30). For exercise versus no treatment (five trials) the WMD was also significantly in favor of exercise (-9.27, 95*% *CI -17.00 to -1.55). Further, they found positive and significant effects for disability. For exercise versus minimal care (14 trials), the WMD was -6.41 (95*% *CI -9.76 to -3.05), and for exercise versus no treatment (9 trials), the WMD was -3.31 (95*% *CI -4.83 to -1.79).

Ferreira *et al. *[22] also explored between-trial variability by meta-regression analyses, and found that only dosage was significantly associated with effect size in pain. 'Dosage' means number of exercise hours and sessions, and the results suggest that for each additional exercise session, the effect size would increase by 0.13 (95*% *CI 0.02 to 0.24) on a 100-point scale. There was no evidence for the effect of exercises on pathogenesis.

### Neck pain

Although there is no general agreement on the classification of NP, the Task Force on Neck Pain and its Associated Disorders [27] suggested a classification of NP severity from grade 1 (no signs or symptoms suggestive of major structural pathology and no or minor interference with activities of daily living; will probably respond to minimal intervention such as reassurance and pain control; does not require intensive investigations or ongoing treatment) to grade IV (signs or symptoms of major structural pathology, such as fracture, myelopathy, neoplasm, or systemic disease; requires prompt investigation and treatment). Most people with NP do not recover completely, and between 50% and 85% of those who experience NP at some initial point will report it again 1 to 5 years later [28].

Leaver *et al. *[21] reported pooled outcomes from three trials showing significant reductions in pain (on a scale of 0 to 100) at the conclusion of a course of specific exercises (WMD -12.00, 95*% *CI -22.00 to -2.00). Pooled results from two trials that reported disability outcomes after general strength and conditioning exercise showed no significant difference compared with minimal intervention at the conclusion of treatment (WMD 1.00, 95*% *CI -3.00 to 5.00). We were not able to identify any reviews exploring between-trial heterogeneity or the effect of exercises on pathogenesis.

### Shoulder pain

SP and shoulder disorders cover a broad spectrum of pain, symptoms, and diagnoses, including tendonitis, rotator-cuff disease, sub-acromial pain, impingement syndrome, and repetitive strain injury. As with NP, there is no general agreement on the classification of painful shoulder conditions [29]. Clinically, patients often present with SP together with stiffness, reduced range of motion, and/or with pain, or other symptoms radiating to the more proximal part of the arm.

Marinko *et al. *[20] included three studies on SP with pain as an outcome, and compared the effects of exercise versus other treatments on pain levels. Data were pooled by use of a random-effects model, which showed an overall SMD in favor of the exercise intervention (SMD -0.30; 95*% *CI -0.48 to -0.12). Four studies included function as an outcome, and compared the effect of exercise versus an alternative intervention or no intervention. Data were pooled by use of a random-effects model, with an overall SMD of 0.15 (95*% *CI 0.01 to 0.29), indicating a minimal positive effect over no care or alternative interventions. We were not able to identify any reviews exploring between-trial heterogeneity or the effect of exercise on pathogenesis.

### Osteoporosis

OP is characterized by a decrease in bone mineral density (BMD), resulting in an increased risk of fracture. The diagnostic criterion for OP is defined as BMD of more than 2.5 standard deviations below the mean bone density of young adult women, whereas the term 'established osteoporosis' includes the presence of a fragility fracture [30]. Clinically, OP is recognized by characteristic fractures after minimal traumas, most often in the hip, vertebrae, or distal forearm. Clinical consequences of OP fractures include pain and physical disability, with loss of independence and need for long-term care as a consequence. Increased mortality is also seen as a direct result of fracture.

In the Cochrane review 'Exercise for preventing and treating osteoporosis in postmenopausal women', Howe *et al. *[15] identified 43 RCTs with more than 4000 participants. None of the studies included pain or physical function as an outcome. Concerning disease pathogenesis in terms of BMD, meta-analyses from 24 RCTs with 1,441 participants showed significant differences in favor of exercise for percentage change in BMD at the spine (MD 0.85; 95*% *CI 0.62 to 1.07), and trochanter (10 RCTs with 815 participants; MD 1.03; 95*% *CI 0.56 to 1.49) for the comparison of any exercise to any non-exercise control. Thus, there is strong evidence for the effect of exercise on the pathogenesis of OP, which was also confirmed by Pedersen and Saltin [23]. When exploring the most effective intervention for BMD at the spine, Howe *et al. *[15] found a combination of exercise programs (MD 3.22; 95*% *CI 1.80 to 4.64) to be most effective, whereas non-weight-bearing high force exercise such as progressive resistance strength training for the legs was most effective for the neck of the femur (MD 1.03; 95*% *CI 0.24 to 1.82). Prevention of fractures is a main aim in management strategies for OP, and Howe *et al. *[15] reported that the risk of fracture in exercise groups was not significantly different from that in controls (OR 0.61; 95*% *CI 0.23 to 1.64 for four RCTs and 539 participants).

### Osteoarthritis

OA is most frequently seen in the hands, hips, and knees, and is characterized by joint pain, stiffness, and functional limitations. From being thought of as a simple degenerative disease, there is now increasing evidence for the involvement of inflammation in OA [31]. The term 'symptomatic OA' is used when both joint-related symptoms and radiographic signs are present [32].

Although evidence-based recommendations for the treatment of OA consider exercise as a cornerstone of modern OA management, the research evidence from clinical trials in hand OA is very limited and conflicting [33]. For hip OA, Fransen *et al. *[17] included 5 RCTs with 204 patients, and reported a small, non-significant effect of exercise on pain in patients with hip OA (SMD -0.33, 95*% *CI -0.84 to 0.17). For physical function, the effect was also small and non-significant (SMD -0.10, 95*% *CI -0.51 to 0.32). The amount of research evidence for knee OA is substantially larger. Fransen *et al. *[16] pooled the results from 32 RCTs with more than 3,600 patients, and reported a beneficial effect of exercise with a SMD of -0.40 (95*% *CI -0.30 to -0.50) for pain; and SMD of -0.37 (95*% *CI -0.25 to -0.49) for physical function. Interestingly, sub-group analyses showed that studies evaluating programs providing at least 12 direct supervision occasions produced significantly greater effects than programs providing less than 12 supervised sessions for both pain ((SMD -0.46; 95*% *CI -0.60 to -0.32) versus SMD -0.28 (95*% *CI -0.40 to -0.16)) and function ((SMD -0.45; 95*% *CI -0.62 to -0.29) versus SMD -0.23 (95*% *CI -0.37 to -0.09)].

According to Pedersen and Saltin [23], there is no evidence for the effect of exercise on the pathogenesis of OA.

### Rheumatoid arthritis

RA is the most common inflammatory rheumatic joint disease. The systemic inflammation leads to synovitis and bone erosion, primarily affecting the small joints of hands and feet, but the larger joints can also be involved. Further, RA is associated with extra-articular features and substantial co-morbidity, and a considerably increased risk of cardiovascular disease (CVD) has been reported [34]. The most typical clinical features of RA are joint pain, stiffness, and reduced physical functioning.

In their Cochrane review of dynamic exercises for RA, Hurkmans *et al. *[18] identified eight RCTs in total. For short-term aerobic capacity and muscle-strength training, statistical pooling of two trials (74 patients) showed a non-significant trend for a positive effect of exercise on functional ability (SMD -0.40, 95*% *CI -0.86 to 0.06), and data from one trial (50 patients) indicated a similar effect on pain (SMD -0.53, 95*% *CI -1.09 to 0.04). For short-term aerobic exercise only, the meta-analysis showed SMD of -0.27 (95*% *CI -0.79 to 0.26) for the effects of exercise on pain (1 trial with 56 patients), and SMD of -0.03 (95*% *CI -0.46 to 0.51) for functional ability (2 trials with 66 patients). We were not able to identify any analyses exploring the effects of particular components of exercise on the effect on clinical outcomes, and there is no evidence for the effect of exercise on the pathogenesis of RA [23].

### Ankylosing spondylitis

AS is a chronic inflammatory rheumatic disease predominantly affecting the axial skeleton and the sacroiliac joints. The main clinical characteristics are pain, stiffness, and loss of spinal mobility, caused by inflammation and damage of spinal structures [35]. Other common features of AS are peripheral arthritis, enthesitis, and anterior uveitis, and recent research has also shown that AS is associated with an increased risk of CVD[36]. Radiographic sacroiliitis has traditionally been the diagnostic hallmark of AS, but the absence of definite radiographic sacroiliitis in the early disease stage has resulted in delays in diagnosis. Efforts have therefore been made to facilitate an early, pre-radiological diagnosis of axial spondyloarthritis [37].

When comparing home exercises with no intervention (1 trial with 155 patients), Dagfinrud *et al. *[19] found a significant positive effect of exercise on pain for patients with AS (SMD 0.49; 95*% *CI 0.17 to 0.81), and a non-significant effect on function (SMD 0.12; 95*% *CI -0.20 to 0.43). There is, to our knowledge, no evidence for the effect of exercise on the pathogenesis of AS, or whether particular components of exercise can improve clinical outcome.

## Discussion

In this overview of systematic reviews on ET for muscle and bone health, we included 9 systematic reviews with a total of 224 trials and 24,059 patients. Overall, we found a substantial amount of empirical evidence supporting ET as a mainstay in the management of MSCs. However, there are differences in the number of included trials between the diagnostic groups included in the present overview. For common conditions such as chronic non-specific LBP and knee OA, there is a substantial number of trials with relatively consistent results showing that exercise has small to moderate beneficial effects on pain and function. For others, such as the inflammatory joint diseases (RA and AS), there are only one or two trials comparing exercises with non-exercise or minimal exercise interventions. When comparing the effect sizes on pain and physical functioning, there seems to be a trend towards larger effects of exercise on pain. The effect sizes were generally larger for pain than for physical function, with significant positive effects for pain in six conditions and for physical function in five conditions. This may indicate that there is no linear relationship between symptoms and functioning, and it may suggest that a relatively large reduction in symptoms is needed to improve physical function. There are also several other important components influencing function, such as environmental and personal factors.

We also intended to explore the evidence for the effect of ET on disease pathogenesis. However, we were not able to identify evidence for the influence of pathogenesis in MSCs, except for OP. Although the disease mechanisms for the common MSCs are largely unknown, there seems to be a gap in the understanding of why ET improves clinical outcome. From a clinical point of view, the most interesting question is 'which exercises for which patients?'. We therefore also explored whether particular components of exercise programs are associated with the size of the treatment effects. Because we did not include single trials comparing different exercise interventions, we could only identify indirect comparisons from the reviews. For LBP and knee OA, for which the substantial number of trials allowed explorative sub-group or meta-regression analyses, the results suggest that 'more is better'; that is, that the treatment effect increases with the number of exercise sessions. However, it is unclear whether the estimated increase is of clinical relevance.

One previous overview has addressed the effect of ET for MSCs [12]. Dziedzic *et al. *also concluded that ET is a beneficial component of the management of MSCs because it reduces pain and disability, and that there is limited evidence for the benefit of one particular approach to exercise over another. However, they also emphasized one important clinical caveat, namely that there is evidence that exercise should not be recommended for acute LBP [12], which was also supported by an updated systematic review [38].

There are several methodological challenges in summarizing evidence from systematic reviews only. There is a substantial number of new trials published in this field every year, and systematic reviews published some years ago may therefore not be based on the most up-to-date evidence. We therefore intended to include Cochrane reviews, because these should be regularly updated. However, this overview clearly shows that this is not the case. We excluded three out of nine eligible Cochrane reviews because they had not been updated after 2007. The decision to exclude Cochrane reviews that had not been updated during the previous five years was arbitrarily chosen and is open to debate. However, considering the substantial number of new trials that have been published on this topic in the past few years, we would suggest that including reviews that are more than 5 years old would not reflect the most up-to-date evidence. Although we performed extensive literature searches, the selection of the three non-Cochrane reviews can also be questioned. However, other systematic reviews on the effects of exercises for LBP, NP and SP also found more or less similar results. For LBP, Macedo *et al. *[39] systematically reviewed 14 RCTs on the effects of motor control exercises for persistent LBP. Their pooled estimates for the comparison between motor control exercise and minimal intervention in reducing pain and disability in both the short and long term were higher than those in the review of Ferreira *et al. *[21]. van Middelkoop *et al. *[40] provided an overview on the effects of ET in patients with chronic LBP; they included 37 RCTs that compared exercise with usual care and found that ET improved post-treatment pain intensity and disability. They also found no evidence that one particular type of ET was clearly more effective than others. In a review of nine trials on the effects of various types of exercise for prevention and cure of non-specific NP in office workers, Sihawong *et al. *[41] found strong evidence for the effects of muscle strengthening and endurance exercises in treating NP. Moderate evidence supported the use of muscle endurance exercise in reducing disability attributed to NP. Teasell *et al. *[42] reviewed the strength of evidence supporting various therapies for whiplash-associated disorder (WAD). They included 40 trials, and found that exercise and mobilization programs for acute and chronic WAD had the strongest supporting evidence, although many questions remain about the relative effects of various protocols. For SP, a recent systematic review of four RCTs examining the effects of exercise for rotator-cuff tendinopathy concluded that the available literature was supportive that exercise reduced pain and functional disability [43]. In a systematic review from 2011, Brudvig *et al. *[44] summarized the published research evidence on the effects of therapeutic exercise and joint mobilization compared with therapeutic exercise alone in patients with shoulder dysfunction. They found no evidence for the beneficial effects of the combination of therapeutic exercise and joint mobilization versus therapeutic exercise alone for reducing pain and increasing function.

An interesting finding of the present study is that Cochrane reviews seem to be of higher methodological quality than non-Cochrane reviews, which is also consistent with reviews specifically addressing this issue [45,46]. Because reviews provide limited information about included trials, the conclusions from tertiary research (overview of systematic reviews) may become too general to be considered as clinically relevant. However, we suggest that findings from overviews can be valuable as a 'compass' for the clinician, and that the decision on the type, dose, and timing of intervention should be shared between the clinician and patient at each clinical encounter. Another limitation related to clinical relevance is the magnitude of the estimated effect sizes. Using a traditional and very rough definition, SMDs between 0.30 and 0.65 can be considered as small to moderate. However, whether the effects are clinically worthwhile is a complex question that should also include the patient's perceptions of risks and costs with the actual treatment. The definition of relatively broad diagnostic groups used in the present overview might also be considered as a limitation from a clinician's point of view. For the regional pain syndromes (LBP, SP, and NP) it is obvious that one size does not fit all; that is, that the effect of ET might not be equal across diagnostic sub-groups. For example, for back pain a recent randomized trial compared stratification of the management based on the patient's prognosis with non-stratified current best practice, and found that stratified care produced disease-specific and general health benefits [47].

Although few reviews addressed potential adverse effects (AEs), exercise for people with MSCs is generally safe and well tolerated, but patients may report minor AEs of exercise such as pain and discomfort [48]. For OP, Howe *et al. *[15] found that fractures and falls were reported as AEs in some studies, but there was no significant effect on the numbers of fractures. By contrast, results from a systematic review showed that exercise can reduce falls, fall-related fractures, and several risk factors for falls in individuals with low BMD [49]. In general, there are few contraindications to prescription of exercise, but co-morbidities should be considered. When prescribing ET as part of the disease management, the exercise program must be individually adopted and targeted depending on the disease severity of the individual patient, and on their physical fitness and any co-morbid conditions. A number of co-morbidities may have an influence on the burden and prognosis of MSCs; in particular, inflammatory rheumatic diseases are associated with a considerably increased risk for CVD [36].

## Conclusions

In conclusion, the present overview shows that ET can decrease pain and improve physical functioning, but there were substantial differences in the amount of research evidence across the included diagnostic groups. For example, the relevant comparison for knee OA included 32 trials with more than 3,600 patients, whereas the results for RA and AS are based on one or two trials with between 50 and 150 patients. Consequently, the pooled estimates for knee OA, LBP, FM, and SP showed consistently significant effects in favor of exercise for both outcomes, whereas for NP, hip OA, RA, and AS, the effect estimates were generally smaller and not always significant. However, for the management of all MSCs included in the present overview, ET is unanimously recommended.

There are, however, important limitations when it comes to implications for clinical practice and research. Firstly, for prescription of exercise programs with optimal health benefits for the individual patient, more knowledge is needed on which particular elements and modes of ET, as well as the doses and frequency of delivery, can improve the outcomes of interest. Secondly, knowledge of the potential influence of ET on disease parthenogenesis and the long-term effects on disease progression is currently very limited. Thirdly, it is still an open question whether the magnitude of the positive effects is clinically worthwhile and whether ET is a cost-effective intervention.

## Competing interests

The authors declare that they have no competing interests.

## Authors' contributions

All authors contributed to the planning of this review, and all participated in the different phases of the trial-selection process. GS and KBH extracted data, and KBH wrote the first draft of the article. All authors read and provided feedback on the draft versions of the article. All the authors have read and approved the final version. KBH is the guarantor.

## Note

* = included reviews

## Pre-publication history

The pre-publication history for this paper can be accessed here:

http://www.biomedcentral.com/1741-7015/10/167/prepub

## Supplementary Material

Additional file 1 **The search strategy for MEDLINE**. The search terms and parameter used to identify non-Cochrane reviews published after January 2007. Similar strategies were used for EMBASE, CINAHL, AMED, and PEDro. Click here for file

Additional file 2 **Excluded Cochrane reviews**. Relevant Cochrane reviews not included in the overview, and reasons for their exclusion. Click here for file
