# Supplementary material for: Exercise therapy for bone and muscle health: an overview of systematic reviews
Source: BMC Med. 2012 Dec 19;10:167. doi: 10.1186/1741-7015-10-167 (PMC3568719; doi:10.1186/1741-7015-10-167)
Supplement: Additional file 1 — The search strategy for MEDLINE. The search terms and parameter used to identify non-Cochrane reviews published after January 2007. Similar strategies were used for EMBASE, CINAHL, AMED, and PEDro. [file 1741-7015-10-167-S1.DOCX]

**Additional file 1:** The search strategy for Medline.

**Description:** The search terms and parameter employed to identify non-Cochrane reviews published after January 2007. Similar strategies were used in Embase, Cinahl, Amed, and PEDro.

1. exp Arthritis/

2. exp Rheumatic Diseases/

3. (felty$ adj2 syndrome).tw.

4. (caplan$ adj2 syndrome).tw.

5. (sjogren$ adj2 syndrome).tw.

6. (sicca adj2 syndrome).tw.

7. still$ disease.tw.

8. bechterew$.tw.

9. ankylosing spondylitis.tw.

10. rheuma$.tw.

11. osteoarthr$.tw.

12. arthritides.tw.

13. (arthritis or arthrosis or arthroses).tw.

14. spondylitis.tw.

15. spondylarthritis.tw.

16. spondylodiskitis.tw.

17. spondyloarthritis.tw.

18. fibromyalgia.tw.

19. gout.tw.

20. or/1-19

21. exp Exercise/

22. exp Exercise Therapy/

23. exp Exercise Movement Techniques/

24. exercise$.tw.

25. physiotherap$.tw.

26. physical therap$.tw.

27. training.tw.

28. or/21-27

29. back.tw.

30. lumbar.tw.

31. lumbago.tw.

32. spine.tw.

33. or/29-32

34. 20 and 33

35. exp Back Pain/

36. back pain.tw.

37. or/35-36

38. 34 or 37

39. 38 and 28

40. limit 39 to "reviews (maximizes specificity)"

41. limit 39 to systematic reviews

42. 40 or 41

43. limit 42 to yr="2007 -Current"

44. shoulder$.tw.

45. 20 and 44

46. Shoulder Pain/

47. shoulder pain.tw.

48. or/46-47

49. 45 or 48

50. limit 49 to "reviews (maximizes specificity)"

51. limit 49 to systematic reviews

52. 50 or 51

53. limit 52 to yr="2007 -Current"

54. exp Cervical Vertebrae/

55. neck.tw.

56. 54 or 55

57. 20 and 56

58. Neck Pain/

59. neck pain.tw.

60. 58 or 59

61. 57 or 60

62. limit 61 to "reviews (maximizes specificity)"

63. limit 61 to systematic reviews

64. 62 or 63

65. limit 64 to yr="2007 -Current"

66. or/43,53,65
