# Supplementary material for: Exercise therapy for bone and muscle health: an overview of systematic reviews
Source: BMC Med. 2012 Dec 19;10:167. doi: 10.1186/1741-7015-10-167 (PMC3568719; doi:10.1186/1741-7015-10-167)
Supplement: Additional file 2 — Excluded Cochrane reviews. Relevant Cochrane reviews not included in the overview, and reasons for their exclusion. [file 1741-7015-10-167-S2.DOCX]

**Additional file 2**

**Title:** Excluded Cochrane reviews.

**Description:** Relevant Cochrane reviews not included in the overview and reasons for exclusion.

*Excluded because of intervention (not land based exercise therapy):*

Bartels EM, Lund H, Hagen KB, Dagfinrud H, Christensen R, Danneskiold-Samsøe B. Aquatic exercise for the treatment of knee and hip osteoarthritis. Cochrane Database of Systematic Reviews 2007, Issue 4. Art. No.: CD005523. DOI: 10.1002/14651858.CD005523.pub2

Dahm KT, Brurberg KG, Jamtvedt G, Hagen KB. Advice to rest in bed versus advice to stay active for acute low-back pain and sciatica. Cochrane Database of Systematic Reviews 2010, Issue 6. Art.No.: CD007612. DOI: 10.1002/14651858.CD007612.pub2.

*Excluded because of outcomes (other than pain and function):*

Choi BKL, Verbeek JH, Tam WWS, Jiang JY. Exercises for prevention of recurrences of low-back pain. Cochrane Database of Systematic Reviews 2010, Issue 1. Art. No.: CD006555. DOI: 10.1002/14651858.CD006555.pub2.

Silva KNG, Mizusaki Imoto A, Almeida GJM, Atallah ÁN, Peccin MS, Fernandes Moça Trevisani V. Balance training (proprioceptive training) for patients with rheumatoid arthritis. Cochrane Database of Systematic Reviews 2010, Issue 5. Art. No.: CD007648. DOI: 10.1002/14651858.CD007648.pub2

*Excluded because of other diagnoses:*

Heintjes EM, BergerM, Bierma-Zeinstra SMA, Bernsen RMD, Verhaar JAN, Koes BW. Exercise therapy for patellofemoral pain syndrome. Cochrane Database of Systematic Reviews 2003, Issue 4. Art. No.: CD003472. DOI: 10.1002/14651858.CD003472.

Pennick V, Young G. Interventions for preventing and treating pelvic and back pain in pregnancy. Cochrane Database of Systematic Reviews 2007, Issue 2. Art. No.: CD001139. DOI: 10.1002/14651858.CD001139.pub2.

Takken T, Van Brussel M, Engelbert RH, van der Net JJ, Kuis W, Helders PPJM. Exercise therapy in juvenile idiopathic arthritis. Cochrane Database of Systematic Reviews 2008, Issue 2. Art. No.: CD005954. DOI: 10.1002/14651858.CD005954.pub2.

Voet NBM, van der Kooi EL, Riphagen II, Lindeman E, van Engelen BGM, Geurts ACH. Strength training and aerobic exercise training for muscle disease. Cochrane Database of Systematic Reviews 2010, Issue 1. Art. No.: CD003907. DOI: 10.1002/14651858.CD003907.pub3.

*Excluded because not updated after January 2007:*

Green S, Buchbinder R, Hetrick SE. Physiotherapy interventions for shoulder pain. Cochrane Database of Systematic Reviews 2003, Issue 2. Art. No.: CD004258. DOI: 10.1002/14651858.CD004258.

Han A, Judd M, Welch V, Wu T, Tugwell P, Wells GA. Tai chi for treating rheumatoid arthritis. Cochrane Database of Systematic Reviews 2004, Issue 3. Art. No.: CD004849. DOI: 10.1002/14651858.CD004849.

Hayden J, van TulderMW, Malmivaara A, Koes BW. Exercise therapy for treatment of non-specific low back pain. Cochrane Database of Systematic Reviews 2005, Issue 3. Art. No.: CD000335. DOI: 10.1002/14651858.CD000335.pub2.

Kay TM, Gross A, Goldsmith CH, Hoving JL, Brønfort G. Exercises for mechanical neck disorders. Cochrane Database of Systematic Reviews 2005, Issue 3. Art. No.: CD004250. DOI: 10.1002/14651858.CD004250.pub3.
